# Cortical Deafness in Children: Scoping Review and Case Report of a Bilateral Perinatal Stroke

**DOI:** 10.1177/08830738241308611

**Published:** 2025-01-08

**Authors:** Francy Cruz-Sanabria, Carolina Ragoni, Renata Salvadorini, Rosa Pasquariello, Emanuele Bartolini, Silvia Paese, Deianira Rinaldi, Francesca Forli, Andrea Guzzetta, Simona Fiori

**Affiliations:** 1 Department of Developmental Neuroscience, IRCCS Stella Maris Foundation, Pisa, Italy; 2 Tuscany PhD Program in Neurosciences, Florence, Italy; 3 Department of Clinical and Experimental Medicine, 9310 University of Pisa, Pisa, Italy; 4 Department of Surgical, Medical and Molecular Pathology and Critical Care Medicine, 9310 University of Pisa, Pisa, Italy; 5 27106 Department of Clinical Science, Intervention and Technology (CLINTEC), Karolinska Institute, Clintec, Hearing Implant Section, Stockholm, Sweden; 6 Neuroscience and Human Genetics Department, Meyer Children's Hospital IRCCS, Florence, Italy

**Keywords:** cortical deafness, perinatal stroke, cerebral palsy, case report, communication, rehabilitation

## Abstract

**Background:**

Persistent cortical deafness in the pediatric population is rarely reported, and there is limited information on its implications for early intervention.

**Objectives:**

This study aims to (1) conduct a scoping review on pediatric cortical deafness and (2) present a case report of a 7-year-old girl with left unilateral spastic cerebral palsy and cortical deafness resulting from presumed perinatal bilateral stroke.

**Methods:**

A search of PubMed, Scopus, and Web of Science identified 407 manuscripts. After the screening, 5 studies met the inclusion criteria for analysis. The case report details clinical characteristics, diagnostic challenges, and intervention strategies for pediatric cortical deafness.

**Results:**

The scoping review highlighted the limited literature on pediatric cortical deafness, emphasizing its association with extensive bilateral lesions and heterogeneous etiology. The case report underscored the need for comprehensive auditory function measurements, early diagnosis, and tailored interventions.

**Conclusions:**

Early and tailored interventions are crucial for improving prognosis in pediatric cortical deafness, particularly in cases associated with bilateral perinatal stroke.

Cortical deafness is a severe impairment in auditory perception at the cerebral cortex level, despite normal peripheral transmission to the brainstem.^1^ Auditory pathways can be studied clinically by otoacoustic emissions for cochlear function, auditory brainstem responses for inner ear, cochlear nerve, and brainstem auditory pathways, and auditory evoked potentials for the electrophysiological responses of the auditory cortex. In cortical deafness, otoacoustic emissions and auditory brainstem responses are normal, whereas abnormal auditory evoked potentials indicate a central origin of the damage.^2^ The bilateral projection of each ear to the brainstem, thalamus, and cortex provides an intrinsic redundancy of the auditory system, which typically protects functional consequence of unilateral brain lesions. Consequently, cortical deafness often results from extensive or “bottleneck” lesions affecting both lemniscal pathways in the brainstem or the auditory cortex bilaterally.^2^ The predominant etiology of cortical deafness is linked to acquired stroke, especially those affecting the temporal cortex and the Heschl gyrus bilaterally, acquired either simultaneously or sequentially. Other structures within the primary auditory network, including subcortical and brainstem structures, may also contribute to cortical deafness when affected bilaterally or in a crossed pattern, such as damage to one cortical hemisphere and the opposite subcortical region.^1^

Clinically, cortical deafness is the most severe form of central hearing impairment spectrum, which also includes auditory verbal agnosia, nonverbal auditory agnosia, receptive amusia, and central auditory processing disorder.^1,2^ Adults with acquired cortical deafness are typically unable to perceive both auditory verbal and nonverbal sounds, yet they retain language skills such as speaking, reading, and writing.^3^ Accurate diagnosis of cortical deafness is crucial for determining prognosis and devising effective rehabilitation strategies.^4^ Although some adults with stroke-related cortical deafness due to transient ischemia or secondary involvement of auditory areas may regain some functional communication, those with bilateral damage to the auditory cortices face similar challenges to other central sensory deficits, such as cortical blindness. These individuals generally do not respond to auditory-verbal rehabilitation and therapy-induced plasticity,^1^ though they may benefit from nonauditory rehabilitation. Accurate and early diagnosis is important for guiding interventions, particularly in pediatric cases, where therapy-induced plasticity may have a broad impact.

Although stroke-related cortical deafness in adults is rare, its symptoms are relatively recognizable and interpretable. However, there is limited understanding of how similar injuries affect early stages of development, especially in infants before language emerges.^4^ A recent systematic review by Silva et al^1^ described 46 clinical cases of stroke-associated cortical deafness in adults aged 21-82 years, with no cases reported in children. The absence of reports on persistent cortical deafness in children may be attributable to greater brain plasticity early in development or, conversely, to limited awareness of this rare condition in diagnostic contexts. The present study has 2 objectives: (1) to perform a scoping review on cortical deafness in the pediatric population, and (2) to present a case report on pediatric cortical deafness likely caused by perinatal injury in a girl with congenital hemiplegia. By examining this case, we aim to enhance understanding of pediatric cortical deafness, including its clinical manifestations, diagnostic challenges, and potential implications for early intervention and communication-focused rehabilitation.

## Methodology

The present study was written in accordance with the relevant EQUATOR Network reporting guidelines, in particular, the Preferred Reporting Items for Systematic Reviews and Meta-Analyses extension for Scoping Reviews (PRISMA-ScR)^5^ and the CARE Guidelines: Consensus-based Clinical Case Reporting Guideline Development.^6^ The systematic search was conducted in PubMed, Scopus, and Web of Science databases to identify relevant articles addressing cortical deafness in the pediatric population (sources were last searched or consulted on data: 20/02/2024). The search strategy employed the following terms: *((“Hearing Loss, Central"[MeSH Terms] OR “Cortical Deafness"[Title/Abstract] OR “Central Deafness"[Title/Abstract]) AND (“Child"[MeSH Terms] OR “Infant, Newborn” [MeSH Terms] OR Adolescent [MeSH Terms]).* Articles were filtered using the following inclusion criteria: type of article: case reports, clinical study, clinical trial; participants: child (birth–18 years), newborn (birth–1 month), infant (birth–23 months), infant (1-23 months), preschool child (2-5 years), child (6-12 years), adolescent (13-18 years). Manuscripts describing cases of inability to understand verbal and nonverbal sounds were considered eligible. Cases describing other types of central hearing impairment, like auditory verbal agnosia (pure word deafness), nonverbal auditory agnosia, receptive amusia, and central auditory processing disorder, were excluded. In addition, articles outside the defined age range and non–English-language publications were excluded. We used data collection forms to ensure comprehensive data capture. Data charting was performed independently by 2 reviewers to enhance reliability and minimize bias. Any discrepancies in data extraction were resolved through consensus discussions between reviewers, with a third reviewer available to adjudicate if necessary. For each included case, the following data were extracted: sociodemographic (age, gender), etiology/pathology associated with the lesion, location of the lesions, and clinical characteristics of cortical deafness.

## Results

### Scoping Review on Cortical Deafness in Pediatric Population

**Figure 1.**
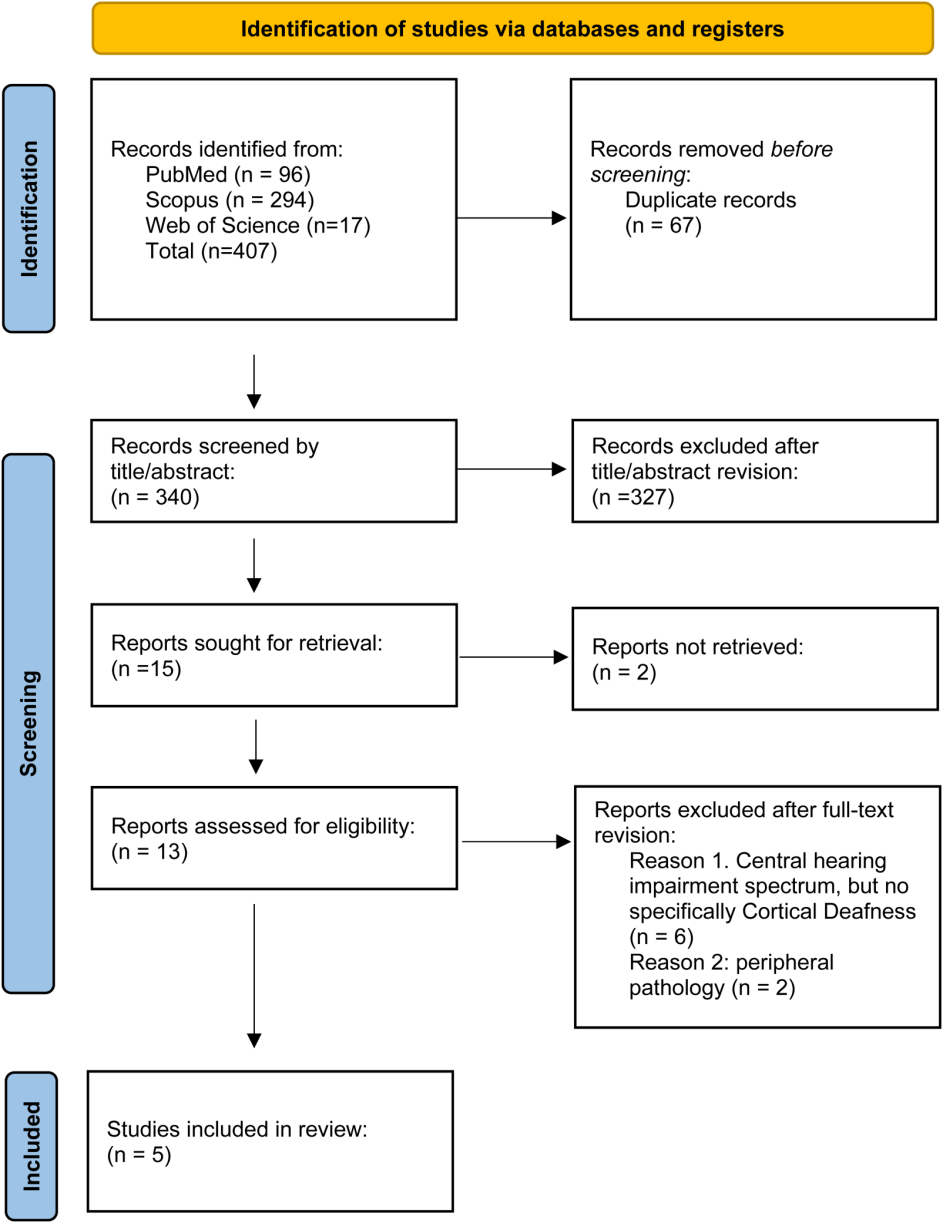
PRISMA flowchart diagram summarizing the selection process.

The systematic search yielded a total of 407 results. After the automatic removal of duplicated manuscripts, 340 articles were screened. Following the initial screening of titles and abstracts, articles not directly addressing the topic of cortical deafness in the pediatric population were excluded. Thirteen manuscripts underwent full-text evaluation, from which 7 articles were excluded. Reasons for exclusion were non–cortical deafness symptoms criteria such as pure word deafness,^7–9^ reduced hearing function,^10^ central auditory deficit associated with deficient interhemispheric transfer,^11^ cochlear implantations suggesting peripheral pathology,^12^ or transient deafness associated with ictal episode^13^; see Table S1 for details. Finally, the total number of included studies was 5.^14–18^ A Preferred Reporting Items for Systematic Reviews and Meta-Analyses (PRISMA) flowchart diagram summarizes the selection and inclusion process (Figure 1). Sociodemographic, clinical, and radiologic characteristics of pediatric cortical deafness cases included in the systematic review are described in Table 1.

**Table 1.** Sociodemographic, Clinical and Radiological Characteristics of Pediatric Cortical Deafness Cases Included in the Systematic Review.

Author, year	Age, gender	Pathology	Lesson topography	Clinical characteristics
Pittet, 2016^15^	11-y-old, M	MELAS (m.3243A > G mitochondrial DNA mutation) syndrome	Bilateral strokelike lesions predominantly affecting the superior temporal lobe, including the primary auditory cortex	Acute profound deafness. Conductive hearing loss was ruled out, and auditory evoked potentials from the brainstem were normal. Despite progressive hearing recovery within 2 weeks, the patient exhibits residual language and cognitive deficits on follow-up
Tonni, 2015^16^	4-y-old, M	Hypoxic-ischemic encephalopathy	Abnormal signal in the lentiform nuclei, thalami, and the Rolandic cortex bilaterally, along with diffuse hyperintensity of the white matter throughout the centrum semiovale bilaterally	Neurodevelopmental follow-up at the age of 4 years showed spastic tetraplegia, seizures, central deafness, and blindness.
Sasidharan, 2020^14^	7-y-old, F	Bacterial meningitis	Not reported	Until the age of 5 months the child used to respond to sounds and suddenly stopped responding, following fever due to acute bacterial meningitis. Absent response to pure-tone audiometry and cortical evoked potentials. Peripheral hearing was normal, as confirmed by ABR and OAEs.
Setzen, 1999^17^	15-mo-old, M	Moyamoya disease (MMD)	Diffuse ischemic damage in subcortical white matter, including areas of temporal lobes. Multiple and focal cortical infarctions in both cerebral hemispheres focused primarily on the frontal, parietal, and temporal areas.	Patient with appropriate developmental milestones, who at age 15 mo presented partial seizures and motor and mental status changes. After that, lack of behavioral response to sound, and absent middle and long latency auditory evoked potentials. Normal peripheral auditory function and brainstem response.
Pillion, 2014^18^	17-y-old, M	Viral meningoencephalitis	Bilateral temporoparietal signal changes reflecting extensive damage to language receptive areas and the first transverse gyrus of Heschl bilaterally. Subcortical damage in the pulvinar region of the thalamus.	A healthy, athletic, 17-y-old boy that acutely developed headaches, fever, and photosensitivity due to presumed meningoencephalitis. An evaluation of auditory processing 6 mo after the initial illness demonstrated absence of response to auditory stimulus, absence of middle-latency response and long-latency response. OAEs and ABR were present, confirming central origin of auditive deficit.

Abbreviations: ABR, auditory brainstem response; MELAS, mitochondrial encephalomyopathy, lactic acidosis, and strokelike episodes; OAEs, otoacoustic emissions.

In cases analyzed, the mean age was 8 years (range: 15 months to 17 years), the majority were males (80%), and causes of cortical deafness were highly variable, including MELAS (m.3243A > G mitochondrial DNA mutation) syndrome,^15^ hypoxic-ischemic encephalopathy due to acute disruption of umbilical cord vessels in a term-survived newborn,^16^ bacterial meningitis,^14^ moyamoya disease,^17^ and viral meningoencephalitis.^18^

Lesion topography was also heterogeneous, but it affected the hemispheres bilaterally in all cases. In the MELAS-associated case, brain MRI revealed multiple and bilateral strokelike lesions predominantly affecting the superior temporal lobe, including both primary auditory cortices.^15^ Abnormal signal in the lentiform nuclei, thalami, and the Rolandic cortex bilaterally, along with diffuse hyperintensity of the white matter throughout the centrum semiovale bilaterally, was observed in the case of hypoxic-ischemic encephalopathy with an MRI performed at 10 and 21 postnatal days.^16^ In moyamoya disease (MMD), a progressive and occlusive cerebrovascular disorder that is predominantly seen in childhood and that predominantly affects the carotid artery system, lesions were described as diffuse ischemic damage in subcortical white matter including areas of the temporal lobes, together with multiple and focal cortical infarctions in both cerebral hemispheres, focused primarily on the frontal, parietal, and temporal areas.^17^ Finally, the case due to viral meningoencephalitis was characterized by bilateral temporoparietal signal changes reflecting extensive damage to language-receptive areas and the first transverse gyrus of Heschl bilaterally, as well as early subcortical damage in the pulvinar region of the thalamus.^18^

Normal peripheral auditory function and brainstem pathway integrity were provided by otoacoustic emission and brainstem auditory evoked potential measurements in most cases,^14,15,17,18^ absent middle and long latency auditory evoked potentials were reported in some cases,^14,17,18^ whereas the behavioral response to sound was reported as absent^14,17,18^ or inconsistent.^15^ On the other hand, in the case described by Tonni et al,^16^ the peripheral auditory function was not described; nevertheless, clinical description indicated that at the age of 4 years, the patient exhibited central deafness, along with spastic tetraplegia, seizures, and blindness.

### Case Presentation

**Figure 2.**
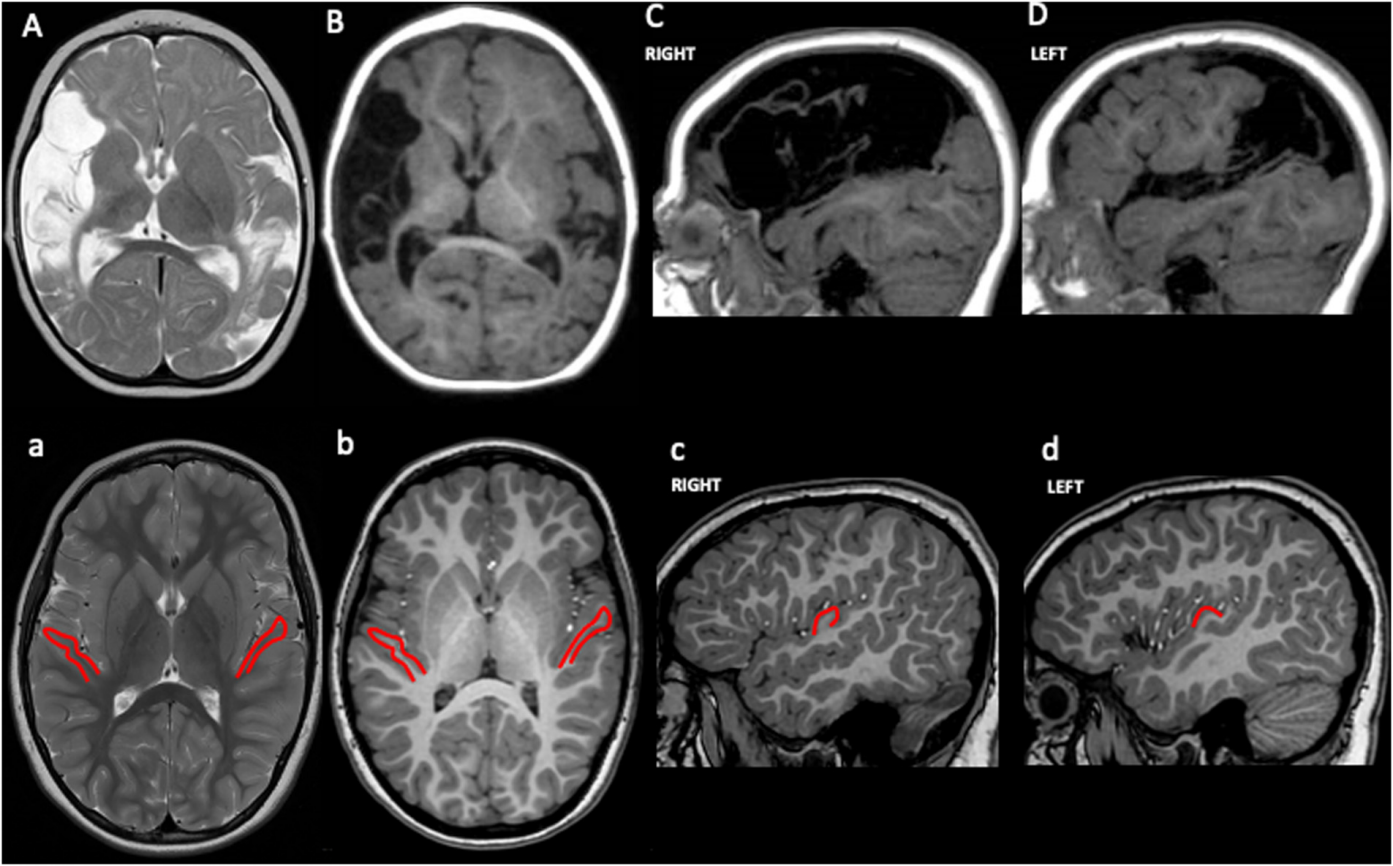
Magnetic resonance imaging of the patient with presumed perinatal bilateral stroke (A–D) and of an age-matched healthy subject (a–d). Axial T2-weighted (A) and axial (B) and sagittal (C, D) T1-weighted images shows malacic and cystic areas with an asymmetrical distribution (right > left) involving cortical territories of middle cerebral artery. Axial T2-weighted (a) and axial (b) and sagittal (c, d) T1-weighted images shows normal anatomy; in-plane projections of Heshl gyrus in the superior temporal gyrus are delineated in red.

We present a 7-year-old female patient with left unilateral spastic cerebral palsy and dysarthria. The family history was unremarkable. She was born following an uneventful pregnancy at 41 weeks’ gestational age through vaginal spontaneous delivery. At birth, she received Apgar scores of 9 and 10 at 1 and 5 minutes, respectively. Her birthweight was 3230 g (50th percentile), length 50 cm (50th-75th percentile), and head circumference 33 cm (25th percentile). Parents initially observed asymmetry in the use of the upper limb at around 4 months of age. A brain magnetic resonance (MR) study was performed at 8 months of age, revealing bilateral strokes extensively involving the superior temporal gyrus and, in the left hemisphere only, extending anteriorly to the central gyrus (Figure 2). Initially, she was referred to a motor rehabilitation program and later, around 2 years of age, to a speech and language auditory-verbal intervention because of a lack of oral language development, with minimal response over the years.

Because of poor communication outcomes, attention deficit, and poor adaptive behavior, comprehensive language and neuropsychological assessments were conducted at 7 years, along with brainstem and nonroutine cortical auditory evoked potentials. Brainstem evoked potentials showed a normal response both for neural conduction and hearing threshold at high frequencies bilaterally, whereas no long-latency potential was elicited. Behavioral audiometry did not provide reliable information on the child's hearing threshold because of poor attention to auditory stimuli.

Functional speech and language assessments for auditory processing revealed adequate relational and nonverbal communication abilities. Comprehension of speech production exhibited a clear discrepancy in behavioral responses between auditory and visual cues. Pure auditory perception displayed inconsistent behavioral changes compared to more consistent responses when visual-articulatory perception of mouth movements was included during verbal communication efforts. Emerging communication pragmatic skills were assessed using the Italian version of the Social Conversational Skills Rating Scale.^19^ Lexical comprehension was measured at the level of 20 months of age using an indirect measure,^20–22^ and at the level of 29 months at direct assessment.^23^ However, because of the characteristics of auditory processing responses, no standardized test for language comprehension and production was deemed reliable. Speech production comprised few words, resulting in poor intelligibility with marked hypernasality. Collectively, these multiple instrumental and clinical characteristics suggested an auditory communication profile compatible with central cortical deafness.

Based on these results supporting a diagnosis of stroke-associated cortical deafness, a visual-gestural 3-week intensive intervention based on sign language was designed to promote communication. The patient demonstrated the capability to learn language signs with 2 to 4 repetitions, depending on sign transparency, to organize morphosyntactic constructions, and to generalize their use with the trainer and primary caregiver, who was also trained. She rapidly achieved generative language proficiency. Through systematically providing reliable communicative input, this personalized habilitative approach dramatically enhanced communication and adaptive behavior in this child.

## Discussion

The case report describes a 7-year-old girl with left unilateral spastic cerebral palsy and dysarthria, diagnosed with cortical deafness due to bilateral strokes affecting the superior temporal gyrus. The case highlights the clinical presentation and management of pediatric cortical deafness. This report, coupled with the scoping review, underscores the rarity and complexity of cortical deafness in the pediatric population.

The scoping review identified only 5 studies meeting the inclusion criteria, confirming the limited research on pediatric cortical deafness. The cases reviewed involved varied etiologies, including mitochondrial encephalopathy with lactic acidosis and strokelike episodes (MELAS) syndrome, hypoxic-ischemic encephalopathy, bacterial meningitis, moyamoya disease, and viral meningoencephalitis. Despite the varied causes, a common feature was extensive bilateral lesions primarily affecting the superior temporal lobe, which includes the primary auditory cortex. This finding aligns with the presented case report, where bilateral strokes in the superior temporal gyrus resulted in cortical deafness.

In our clinical case, extensive instrumental assessments and a tailored visual-gestural intervention were critical in diagnosing and managing cortical deafness. To our knowledge, this is the first report of cortical deafness due to presumed perinatal brain injury in a girl with unilateral spastic cerebral palsy. Brain MRI conducted before the child's first year of life indicated the presence of bilateral strokes with asymmetrical extension. Both strokes affected the superior temporal gyrus, including the Heschl cortex, along with the primary sensorimotor cortex on the site contralateral to the observed hemiparesis. Although no specific triggering event was linked to the cerebrovascular event, we posit that it likely occurred either pre-perinatally or within the initial months of the child's life, suggesting a presumed perinatal stroke.^24^ The occurrence of bilateral strokes is relatively common, constituting 24% to 35% of perinatal strokes.^25^

Considering the existing literature on central hearing loss in the pediatric population, the rarity of cortical deafness in children becomes evident. Our case is consistent with findings in the literature, which shows that cortical deafness often results from extensive bilateral lesions. Notably, from the literature review, it emerged that the severity of cortical deafness possibly varies in function of damage extension. For example, in cases of MELAS syndrome with cortical deafness associated with strokelike lesions mainly affecting the superior temporal lobe, some improvement in cortical deafness was seen after 2 weeks.^15^ In contrast, cases of hypoxic-ischemic encephalopathy with extensive bilateral damage to regions like the lentiform nuclei, thalami, Rolandic cortex bilaterally, and centrum semiovale showed a persistent inability to respond to auditory stimuli, even years later.^16^

Our patient exhibited a spectrum of clinical features, including unilateral spastic cerebral palsy and dysarthria, underscoring the complex nature of cortical deafness in the pediatric population. Nevertheless, the appropriate and intensive visual-gestural intervention based on sign language resulted in significant improvements in language proficiency and communication skills. This personalized approach emphasizes the importance of early and tailored interventions in mitigating the impact of cortical deafness on communication and adaptive behavior.

Compared to lesions acquired late, brain damage occurring during early development offers greater potential for superior functional recovery because of heightened brain plasticity. This phenomenon has been extensively substantiated in the context of language function^26–29^ and the corticospinal system governing motor function,^30^ where the location and severity of brain lesions have been correlated with the extent of dysfunction.^31^ However, sensory functions generally show less brain plasticity. Neuroimaging studies reveal that, unlike the remapping observed in the primary motor cortex, somatosensory regions do not undergo such adaptations in the preserved hemisphere following unilateral congenital brain injury.^32–34^ Similarly, impairment of the central visual pathways results in enduring cerebral visual impairment, with limited prospects for the recovery of conscious vision.^35^ Our case report aligns with these findings, underscoring minimal functional recovery following direct damage to the sensory (auditory) cortex. This case report aims to heighten awareness of the possible sensory processing disorders associated with cerebral palsy among health care professionals. Such insights can help refine prognosis, promote early intervention, and support tailored rehabilitation programs for individuals with early brain injuries.

Reports on the prevalence of hearing loss in children with cerebral palsy demonstrate variability, estimated between 12% and 40%.^36,37^ Interestingly, studies exploring hearing abilities in cerebral palsy children often focus on conductive or sensorineural deficits,^38,39^ with little acknowledgment of the potential contribution of cortical auditory impairment. A recent literature review of the evidence specifically focused on the early stages of hearing diagnosis and interventions for infants at high risk, including those with a diagnosis of cerebral palsy, indicates that although hearing screening is similar to that for typically developing infants, diagnosis of hearing loss is often delayed in this clinical population. Moreover, screening measurements include otoacoustic emissions and auditory brainstem responses but not cortical evoked potentials.^40^ These results suggest that cases of cortical deafness could be underestimated and highlights the relevance of including in the screening protocols measurements involving multiple elements of the auditory pathway, from the external ear to the cortex.

In a recent case series involving children with perinatal bilateral stroke, language dysfunction is frequently reported without specific mention of audiologic assessments.^25^ The underestimation of auditory perception in children could detrimentally affect treatment outcomes. As a strength, our case report describes how we conducted a nonroutine evaluation, including cortical evoked potentials that were not elicited and oriented the diagnosis. However, it is crucial to note that there is currently no validated approach to confirm this diagnosis as cortical evoked potentials exhibit limited feasibility and reproducibility during early developmental stages, which may represent a limitation of our case report. For our subject, a comprehensive analysis of deep brain lesions, communication development trajectory, and audiologic evaluations proved fundamental in defining the clinical profile. We advocate for this holistic approach to enhance our understanding of the intricate relationship between altered brain structure and dysfunction, enabling the tailoring of intervention strategies.

The functional diagnosis significantly influences habilitation strategies. Despite cortical deafness being traditionally perceived as resistant to auditory-verbal intervention, our case prompts the recommendation of a bimodal approach that includes both auditory-verbal and visual-gestural techniques. This combined approach is highly beneficial for enhancing communication functioning and adaptive behavior. This suggestion is grounded in the broad time frame for language development during infancy and the distinctive language development trajectory observed in perinatal stroke cases. Likewise, the improved response after bimodal auditory verbal stimulation has been demonstrated in experimental settings.^41^ The specific case we present encountered a diagnostic challenge related to communication atypicality, impacting her communication and adaptive behavior negatively until school age. Additionally, emerging techniques for enhancing cortical brain plasticity, such as transcranial direct current stimulation, have been experimentally applied for conditions like auditory agnosia.^42^

Although our scoping literature review provides insights into cortical deafness in the pediatric population, it is essential to acknowledge certain limitations. Firstly, the limited number of studies meeting the inclusion criteria highlights the scarcity of literature on pediatric cortical deafness, suggesting a gap in understanding and research in this area. Additionally, the heterogeneity among the included studies regarding the reported measures poses challenges in synthesizing findings and drawing definitive conclusions. Additionally, the exclusion of non–English language publications may introduce language bias. Despite these limitations, we believe this review contributes valuable insights into pediatric cortical deafness, emphasizing the need for further research and encouraging standardized methodologies in this field.

## Conclusion

This report presents a rare case of cortical deafness resulting from perinatal bilateral brain injury to the superior temporal gyrus. Cortical deafness in congenital cases is exceptionally rare because of the typically distributed nature of the auditory network. Our literature review identified a limited number of pediatric cortical deafness cases with various etiologies, all involving extensive bilateral lesions in the superior temporal lobe. This case highlights the importance of recognizing cortical deafness as a potential factor in the communication profile of children with cerebral palsy resulting from bilateral brain injury. We recommend early, comprehensive auditory evaluations, including brainstem and cortical auditory evoked potentials—to ensure accurate diagnosis. Our findings also underscore the value of a bimodal intervention approach that combines auditory-verbal and visual-gestural techniques, leveraging early brain plasticity to support communication and adaptive behavior.

## Supplemental Material

sj-docx-1-jcn-10.1177_08830738241308611 - Supplemental material for Cortical Deafness in Children: Scoping Review and Case Report of a Bilateral Perinatal Stroke Supplemental material, sj-docx-1-jcn-10.1177_08830738241308611 for Cortical Deafness in Children: Scoping Review and Case Report of a Bilateral Perinatal Stroke by Francy Cruz-Sanabria, Carolina Ragoni, Renata Salvadorini, Rosa Pasquariello, Emanuele Bartolini, Silvia Paese, Deianira Rinaldi, Francesca Forli, Andrea Guzzetta and Simona Fiori in Journal of Child Neurology
