# Supplementary material for: Cortical Deafness in Children: Scoping Review and Case Report of a Bilateral Perinatal Stroke
Source: J Child Neurol. 2025 Jan 8;40(6):469–77. doi: 10.1177/08830738241308611 (PMC12084659; doi:10.1177/08830738241308611)
Supplement: sj-docx-1-jcn-10.1177_08830738241308611 - Supplemental material for Cortical Deafness in Children: Scoping Review and Case Report of a Bilateral Perinatal Stroke [file sj-docx-1-jcn-10.1177_08830738241308611.docx]

**SUPPLEMENTARY MATERIAL**

Search terms details by Database:

SCOPUS: (TITLE-ABS-KEY("Hearing Loss, Central"OR "Cortical Deafness" OR "Central Deafness") AND TITLE-ABS-KEY ("Child" OR "Infant, Newborn" OR "Adolescent" )) AND ( LIMIT-TO ( DOCTYPE,"ar" ) ) AND ( LIMIT-TO ( LANGUAGE,"English" ) ) AND ( LIMIT-TO ( EXACTKEYWORD,"Human" ) )

294 documents found.

WEB OF SCIENCE: ((TS=("Central Deafness" OR "Cortical Deafness" OR "Hearing Loss, Central")) AND (TS=( Child OR Infant OR Newborn OR Adolescent)))

17 results from Web of Science Core Collection.

PUBMED: (("Hearing Loss, Central"[MeSH Terms] OR "Cortical Deafness"[Title/Abstract] OR "Central Deafness"[Title/Abstract]) AND ("Child"[MeSH Terms] OR "Infant, Newborn" [MeSH Terms] OR Adolescent [MeSH Terms])). Filters: Case Reports, Clinical Study, Clinical Trial, Randomized Controlled Trial, Review, Child: birth-18 years, Newborn: birth-1 month, Infant: birth-23 months, Infant: 1-23 months, Preschool Child: 2-5 years, Child: 6-12 years, Adolescent: 13-18 years.

96 documents found.

**Table S1.** Articles excluded after full-text revision:

| **Author and Title** | **Pathology and clinical characteristics** | **Reason for exclusion after full-text revision** |
| --- | --- | --- |
| Levaux J., 2022.  *Cerebral Seizures in an Adolescent with Jervell and Lange-Nielsen Syndrome: It May Not Be Epilepsy* | Jervell and Lange-Nielsen syndrome associated congenital long QT syndrome (LQTS) in a 13 years-old Female patient. | Cochlear implantations suggest peripheral pathology |
| Leng Y., et al 2013.  *The mitochondrial DNA 10197 G4A mutation causes MELAS/Leigh overlap syndrome presenting with acute auditory agnosia* | Mitochondrial encephalomyopathy with lactic acidosis and stroke-like episodes/Leigh (MELAS/ LS) overlap syndrome in a 14-year-old, male showing multiple lesions involving bilateral temporal lobes, the basal ganglia and the brainstem presenting with acute auditory verbal agnosia (He could not understand voices and words, but could still recognize the sound of music and the ticking of a clock). | Symptoms described suggests a case of Auditory verbal agnosia, belonging to the central hearing impairment spectrum but preserving the ability to perceive nonverbal signals, then not being pure cortical deafness. |
| Ansink 1975.  *A complicated case of cortical deafness in a child*  Tervoort B.T.1979  *Cortical Word Deafness In a Child: A case study* | Not clear etiology. Case of a 14-year-old male slightly retarded in its motor development with an extreme retardation in speech: Tone-audiograms, however, did not reveal any abnormality, but patient was unable to identify verbal sounds, while nonverbal sounds were normally perceived. | Symptoms described suggests a case of " cortical word deafness, belonging to the central hearing impairment spectrum, but preserving the ability to perceive nonverbal signals, then not being pure cortical deafness. |
| Caridi G et.al 2006. *Nephronophthisis type 1 deletion syndrome with neurological symptoms: Prevalence and significance of the association* | Patient 5 affected by Nephronophthisis (NPHP), aged 15 years, presented mild developmental delay, hypotonia, and reduced hearing function from birth. Visual loss was evident at the age of 2 years and the patient became blind by the age of 10 years. Audiometry- and acoustic-evoked potentials demonstrated central hearing loss. | Symptoms described as “reduced hearing function”/ “central hearing loss”, not clearly “cortical deafness symptoms” described. No brain MRI remarkable signs |
| Ghosh D., 2001.  *Ictal deafness - A report of three cases* | Transient ictal deafness observed in three cases of partial seizures. One of them had seizure spread to the temporal lobe to produce a complex partial seizure. Two of them have CT-detected lesions in the left temporal/parietal area. The other one had left temporal focus on EEG with a normal imaging study. | Transient symptoms |
| Bamiou DE., 2007 *Auditory and verbal working memory deficits in a child with congenital aniridia due to a PAX6 mutation* | Case of a 12 year old boy with a known mutation of thePAX6 gene. There were parental concerns regarding his hearing, but repeated pure-tone audiograms were normal. Instead, tests which required auditory interhemispheric transfer showed abnormal results, together with impaired verbal working memory | Central auditory deficit associated to deficient interhemispheric transfer |
| Kaga et al., 2003  *Auditory Agnosia in Children after Herpes Encephalitis* | Herpes encephalitis in four pediatric patients with auditory agnosia whose bilateral auditory cortices were damaged by herpes encephalitis at an early age are presented.  Patients 1 and 3 were indifferent to sounds and could not distinguish any sound. Speech discrimination, perception of environmental sounds and music and auditory comprehension were all totally impaired.  Patient 2 and 4: can hear but cannot distinguish speech, environmental sounds and music. | Although patients were unable to discriminate auditory information, audiograms showed residual hearing, thus indicating cases of Auditory agnosia and not pure cortical deafness. |
